# Supplementary material for: Hospitalizations and length of stay of cancer patients: A cohort study in the Brazilian Public Health System
Source: PLoS One. 2020 May 20;15(5):e0233293. doi: 10.1371/journal.pone.0233293 (PMC7239479; doi:10.1371/journal.pone.0233293)
Supplement: Attachment — Submitted filename: BHSR-D-19-00811_reviewer.pdf [file pone.0233293.s001.pdf]

PLOS ONE

PATIENTS HOSPITALIZATIONS AND LENGTH OF HOSPITAL STAY WITH CANCER TREATMENT BY BRAZILIAN PUBLIC HEALTH SYSTEM

--Manuscript Draft--

**Manuscript Number:**	PONE-D-19-27629
**Article Type:**	Research Article
**Full Title:**	PATIENTS HOSPITALIZATIONS AND LENGTH OF HOSPITAL STAY WITH CANCER TREATMENT BY BRAZILIAN PUBLIC HEALTH SYSTEM
**Short Title:**	A population-based cohort

Corresponding Author: FLAVIA FELICIANA SILVA, Msc
FEDERAL UNIVERSITY DE MINAS GERAIS
BELO HORIZONTE, Minas Gerais BRAZIL

Keywords: oncology; hospitalization; length of stay; hospital service

Abstract:

Abstract

Background

Hospitalizations are part of the course of patients with cancer. In Brazil, the current health care model has proven to be hospital-centred and its impacts on the Public Health System. The objective of this study was to investigate the factors associated with hospitalization and length of stay in cancer patients during the first year of his treatment by Brazilian Public Health System (SUS), for most incidents canceres in the brazilian population.

Methods

In this retrospective observational study, we investigated all patients under oncological treatment in the SUS, between October 1, 2010 and October 31, 2014, with the cancer primary locations: prostate, lung and bronchi, breast, colon and rectum, cervix, and stomach.The analysis of demographic and clinical variables on the chance estimates of hospitalization was performed using the binary logistic regression model to estimate the odds ratio (OR), with 95% confidence interval (95% CI) and p-value for each variable. To evaluate the influence of explanatory variables on the total annual length of hospital stay, we used the negative binomial regression model to estimate the Relative Risk (RR), with 95% confidence interval (95% CI) and p-value for each period variable.

Results

We analyzed 417,477 patients and the hospitalization at least once within the first year of cancer treatment occurred in 34% of them. Regarding the primary tumor location, patients with tumors in the colon and rectum (OR = 4.42; CI: 4.27-4.48) had higher chances of hospitalization. Patients with cancer stage IV more likely to be hospitalized in the order of 62% (OR = 1.62; CI: 1.57-1.70). The factors associated with the risks of higher length of stay were: to be male (RR = 0.98 for women; CI: 0.97-0.99); with younger ages, since each year of age reduced by 1% the risk of hospital stay (RR = 0.99; CI: 0.98-0.99); resident of the North region (RR≤ 0.93 for the other regions); with primary tumor location in the colon and rectum (RR = 1.37; CI: 1.35-1.40); and to present tumor metastasis, stage IV (RR = 1.04; CI: 1.04-1.06). Each occurrence of comorbidity increased by 19% (OR = 1.19; CI: 1.18-1.19) the chance of hospitalization and by 3% (RR = 1.03; CI: 1.02-1.03) the risk of higher length of hospital stay. To be emergency hospitalized increased the risk of higher length of hospital stay by 28% (RR = 1.28; CI: 1.27-1.28).

Conclusion

The results of this study pointed out the importance of rethinking the effectiveness of cancer care in Brazil. For this, many efforts in defense of the SUS will be required to deal with the nowadays organizational structures of public health and its complexity.

Powered by Editorial Manager® and ProduXion Manager® from Aries Systems Corporation

Order of Authors:	FLAVIA FELICIANA SILVA, Msc
	Gisele Macedo da Silva Bonfante
	Hugo André da Rocha
	Agner Pereira Lana
	Mariangela Leal Cherchiglia
**Additional Information:**	
**Question**	**Response**
**Financial Disclosure**<br><br>Enter a financial disclosure statement that describes the sources of funding for the work included in this submission. Review the submission guidelines for detailed requirements. View published research articles from *PLOS ONE* for specific examples.<br><br>This statement is required for submission and **will appear in the published article** if the submission is accepted. Please make sure it is accurate.<br><br>**Unfunded studies**<br>Enter: *The author(s) received no specific funding for this work.*<br><br>**Funded studies**<br>Enter a statement with the following details:<br>• Initials of the authors who received each award<br>• Grant numbers awarded to each author<br>• The full name of each funder<br>• URL of each funder website<br>• Did the sponsors or funders play any role in the study design, data collection and analysis, decision to publish, or preparation of the manuscript?<br>• **NO** - Include this sentence at the end of your statement: *The funders had no role in study design, data collection and analysis, decision to publish, or preparation of the manuscript.*<br>• **YES** - Specify the role(s) played.<br><br>* typeset	The funders had no role in study design, data collection and analysis, decision to publish, or preparation of the manuscript.
**Competing Interests**<br><br>Use the instructions below to enter a competing interest statement for this submission. On behalf of all authors,	The authors have declared that no competing interests exist.

disclose any competing interests that could be perceived to bias this work—acknowledging all financial support and any other relevant financial or non-financial competing interests.<br><br>This statement **will appear in the published article** if the submission is accepted. Please make sure it is accurate. View published research articles from *PLOS ONE* for specific examples.<br><br>**NO authors have competing interests**<br><br>Enter: *The authors have declared that no competing interests exist.*<br><br>**Authors with competing interests**<br><br>Enter competing interest details beginning with this statement:<br><br>*I have read the journal's policy and the authors of this manuscript have the following competing interests: [insert competing interests here]*<br><br>* typeset	
**Ethics Statement**<br><br>Enter an ethics statement for this submission. This statement is required if the study involved:<br><br>• Human participants<br>• Human specimens or tissue<br>• Vertebrate animals or cephalopods<br>• Vertebrate embryos or tissues<br>• Field research<br><br>Write "N/A" if the submission does not require an ethics statement.<br><br>General guidance is provided below. Consult the submission guidelines for detailed instructions. **Make sure that all information entered here is included in the Methods section of the manuscript.**	This study was part of the project “Epidemiological, economic and care paths of high cost procedures in SUS: use of patient-centered database from the integration of health information system records”, approved by the Research and Ethics Committee of the Federal University de Minas Gerais, with Process number: CAAE 44121315.2.0000.5149.

**Format for specific study types**<br><br>**Human Subject Research (involving human participants and/or tissue)**<br>• Give the name of the institutional review board or ethics committee that approved the study<br>• Include the approval number and/or a statement indicating approval of this research<br>• Indicate the form of consent obtained (written/oral) or the reason that consent was not obtained (e.g. the data were analyzed anonymously)<br><br>**Animal Research (involving vertebrate animals, embryos or tissues)**<br>• Provide the name of the Institutional Animal Care and Use Committee (IACUC) or other relevant ethics board that reviewed the study protocol, and indicate whether they approved this research or granted a formal waiver of ethical approval<br>• Include an approval number if one was obtained<br>• If the study involved *non-human primates*, add *additional details* about animal welfare and steps taken to ameliorate suffering<br>• If anesthesia, euthanasia, or any kind of animal sacrifice is part of the study, include briefly which substances and/or methods were applied<br><br>**Field Research**<br><br>Include the following details if this study involves the collection of plant, animal, or other materials from a natural setting:<br>• Field permit number<br>• Name of the institution or relevant body that granted permission	
**Data Availability**<br><br>Authors are required to make all data underlying the findings described fully available, without restriction, and from the time of publication. PLOS allows rare exceptions to address legal and ethical concerns. See the PLOS Data Policy and FAQ for detailed information.	Yes - all data are fully available without restriction

A Data Availability Statement describing where the data can be found is required at submission. Your answers to this question constitute the Data Availability Statement and **will be published in the article**, if accepted.<br><br>**Important:** Stating 'data available on request from the author' is not sufficient. If your data are only available upon request, select 'No' for the first question and explain your exceptional situation in the text box.<br><br>Do the authors confirm that all data underlying the findings described in their manuscript are fully available without restriction?	
**Describe where the data may be found in full sentences. If you are copying our sample text, replace any instances of XXX with the appropriate details.**<br><br>• If the data are **held or will be held in a public repository**, include URLs, accession numbers or DOIs. If this information will only be available after acceptance, indicate this by ticking the box below. For example: *All XXX files are available from the XXX database (accession number(s) XXX, XXX.*).<br>• If the data are all contained **within the manuscript and/or Supporting Information files**, enter the following: *All relevant data are within the manuscript and its Supporting Information files.*<br>• If neither of these applies but you are able to provide **details of access elsewhere**, with or without limitations, please do so. For example:<br><br>*Data cannot be shared publicly because of [XXX]. Data are available from the XXX Institutional Data Access / Ethics Committee (contact via XXX) for researchers who meet the criteria for access to confidential data.*<br><br>*The data underlying the results presented in the study are available*	All relevant data are within the manuscript and its Supporting Information files.

from (include the name of the third party and contact information or URL).<br>• This text is appropriate if the data are owned by a third party and authors do not have permission to share the data.<br><br>* typeset	
Additional data availability information:	Tick here if the URLs/accession numbers/DOIs will be available only after acceptance of the manuscript for publication so that we can ensure their inclusion before publication.

Powered by Editorial Manager® and ProduXion Manager® from Aries Systems Corporation

Click here to download Manuscript MANUSCRIPT.docx

PATIENTS HOSPITALIZATIONS AND LENGTH OF HOSPITAL STAY WITH CANCER TREATMENT BY BRAZILIAN PUBLIC HEALTH SYSTEM

Flávia Feliciana Silva[1], Gisele Macedo da Silva Bonfante[2], Hugo André da Rocha[3], Agner Pereira Lana[4], Mariangela Leal Cherchiglia[5]

[1*]Postgraduate Program in Public Health, Medicine School, Federal University of Minas Gerais, Brazil [2]Department of Odontology, Pontifical Catholic University of Minas Gerais, Brazil [3]Postgraduate Program in Public Health, Medicine School, Federal University of Minas Gerais, Brazil [4]Postgraduate Program in Public Health, Medicine School, Federal University of Minas Gerais, Brazil [5]Department of Preventive and Social Medicine, Medicine School, Federal University of Minas Gerais, Brazil

*flaviafelisil@yahoo.com.br (FFS)

Abstract

Background

Hospitalizations are part of the course of patients with cancer. In Brazil, the current health care model has proven to be hospital-centred and its impacts on the Public Health System. The objective of this study was to investigate the factors associated with hospitalization and length of stay in cancer patients during the first year of his treatment by Brazilian Public Health System (SUS), for most incidents canceres in the brazilian population.

Methods

In this retrospective observational study, we investigated all patients under oncological treatment in the SUS, between October 1, 2010 and October 31, 2014, with the cancer primary locations: prostate, lung and bronchi, breast, colon and rectum, cervix, and stomach.The analysis of demographic and clinical variables on the chance estimates of hospitalization was performed using the binary logistic regression model to estimate the odds ratio (OR), with 95% confidence interval (95% CI) and p-value for each variable. To evaluate the influence of

explanatory variables on the total annual length of hospital stay, we used the negative binomial regression model to estimate the Relative Risk (RR), with 95% confidence interval (95% CI) and p-value for each period variable.

Results

We analyzed 417,477 patients and the hospitalization at least once within the first year of cancer treatment occurred in 34% of them. Regarding the primary tumor location, patients with tumors in the colon and rectum (OR = 4.42; CI: 4.27-4.48) had higher chances of hospitalization. Patients with cancer stage IV more likely to be hospitalized in the order of 62% (OR = 1.62; CI: 1.57-1.70). The factors associated with the risks of higher length of stay were: to be male (RR = 0.98 for women; CI: 0.97-0.99); with younger ages, since each year of age reduced by 1% the risk of hospital stay (RR = 0.99; CI: 0.98-0.99); resident of the North region (RR≤ 0.93 for the other regions); with primary tumor location in the colon and rectum (RR = 1.37; CI: 1.35-1.40); and to present tumor metastasis, stage IV (RR = 1.04; CI: 1.04-1.06). Each occurrence of comorbidity increased by 19% (OR = 1.19; CI: 1.18-1.19) the chance of hospitalization and by 3% (RR = 1.03; CI: 1.02-1.03) the risk of higher length of hospital stay. To be emergency hospitalized increased the risk of higher length of hospital stay by 28% (RR = 1.28; CI: 1.27-1.28).

Conclusion

The results of this study pointed out the importance of rethinking the effectiveness of cancer care in Brazil. For this, many efforts in defense of the SUS will be required to deal with the nowadays organizational structures of public health and its complexity.

Introd[illegible]on

Cancer is among the leading causes of worldwide morbidity and mortality [1]. In Brazil, late stage diagnosis of disease emphasize public health problem [1-3]. Cancer diagnosis at an advanced stages reduces the patient possibility cure for disease, increases their vulnerability to clinical complications and the need for greater technological apparatus during treatment, with consequent economic impact [4].

Hospitalizations are part of the course of patients with cancer [5] and increase the cost of healthcare [6-7], especially in the last days of life (Park and Song, 2018). Cancer treatment hospitalizations cost approximately $3 million annually in Brazil [2], which have a rising trend [8]. During treatment, patients may also have complications related to previous disease, other acute medical conditions or cancer worsening that influence the frequency of hospitalizations and, once admitted, his length of hospital stay [9].

The Brazilian Public Health System (SUS) is organized into healthcare net that guide the flow of users in graduated system. The primary health care is an entry point for SUS and hospitals represent complexity greater level [2]. However, the current health care model has proven to be fragmented, hospital-centred and particular emphasis is being placed on population acute demands [6,10-11].

Hospitals admissions in SUS are regulated by the bed regulation system in accordance with the principles of equal accessibility and universality recognising the positive provision of the right to health. However, the strong component of social inequality in Brazil is reflected in the disparity among available resources. The offer of hospital beds in Brazil is mainly, coming from the private network, through contractual relationships between the public and the private [8].

There is not always proportionality between frequency of hospitalizations and hospital length of stay. A cancer patient may be hospitalized once, but remain hospitalized for a long period of time, while others are hospitalized multiple times with short stay totaling shorter length of stay. Hospitalization and length of stay are relevant health indicators and of cancer patient care quality [6, 9, 12].

Whereas the growing incidence and prevalence of cancer in Brazil and its impact on the health system, the objective of this study was to investigate the factors associated with hospitalization and length of stay in cancer patients during the first year of his treatment by SUS, for most incidents canceres in the brazilian population.

Method

This research consists a retrospective observational study. As data sources, we used the National Oncological Database, a population-based cohort which contains all records of patients under oncological treatment in the SUS, from 2001 to 2015. This data was obtained from the National Database of Health, a database centered on the individual that was built by record linkage techniques used to integrate data from the major SUS Information Systems: Outpatient (SIA), Hospital (SIH) and Mortality (SIM); from 2000 to 2015 [13].

We included patients who started cancer treatment at SUS, between October 1, 2010 and October 31, 2014, with the cancer primary locations: prostate, lung and bronchi, breast, colon and rectum, cervix, and stomach; according to the International Classification of Disease, 10th Revision (ICD-10). Regarding the incidence, these tumors are considered as the greatest magnitude in Brazil [1]. Exclusion criteria were individuals under 19 years of age due their particularities of treatment, authorizations and payments within the SUS; and those patients who underwent only surgery as treatment modality, from whose data available were not complete for all the research variables.

The patients analyzed were followed for one year after the treatment start by hospitalization. The extension of this follow-up period could result in a greater probability of including data from patients already cured of cancer, information that is would not be subject of this study.

The clinical outcomes evaluated were hospitalization and length of stay. The hospitalization variable was observed considering the whole study population, categorized as 'hospitalized' or 'not hospitalized' within the first year after cancer treatment start. For the hospitalized patients, the length of stay (in days) was observed during the period. This variable was measured by the sum of the days between each admission and hospital discharge.

Demographic characteristics included patient's sex, age group (19-24, 25-39, 40-59, 60-79, ≥80) and geographic region of residence (Southeast, Northeast, South, Midwest and North). Clinical characteristics included primary tumor location (prostate, lung and bronchi, breast, colon and rectum, cervix, stomach); cancer stage (I, II, III, IV); comorbidities (0, 1-3,>3); and hospitalization within the first year after treatment start (Yes / No). The cancer stage variable was based on the General Classification of Malignant Tumors, which ranges from 0 to IV. The length of stay analysis also included the variable 'emergency hospitalization' (Yes / No).

The hospitalization causes variable was used only in the descriptive analysis, since it correlates with other clinical patient's characteristics, and any regression model requires that the explanatory variables be independent. The hospitalization causes were observed by the main procedure code performed at the hospital, according the SUS Procedures, Medicines and OPM Table Management System (SIGTAP) [2]. The procedures are grouped in the reference table, as following: 'hospitalizations for oncological surgeries', 'hospitalizations for other surgeries' (non-oncology-specific surgical procedures), 'hospitalizations for radiotherapy and/or chemotherapy', 'hospitalizations due to the clinical complications of cancer treatment' and 'hospitalizations for other clinical causes'.

The 'hospitalizations due to the clinical complications of cancer treatment' are defined as the inpatient treatment regarding the complications of the cancer or due its treatment. Clinical complications comprehend acute complication, predictable or not, due to malignant neoplasm or its treatment, which requires hospitalization. Anemias, myelodysplastic syndromes, nausea and vomiting, pneumonia, hemorrhages, cachexia, sepsis, seizures, among others, are examples of complications included in this classification [2].

Frequency measures were performed to describe the demographic and clinical characteristics of the study population. The analysis of these variables on the chance estimates of hospitalization was performed using the binary logistic regression model to estimate the odds ratio (OR), with 95% confidence interval (95% CI) and p-value for each variable. The logistic regression model used to analyze the association of patients' demographic and clinical characteristics with the hospitalization outcome had its performance evaluated by the Receiver Operating Characteristic (ROC) curve.

To evaluate the influence of explanatory variables on the total annual length of hospital stay, we used the negative binomial regression model to estimate the Relative Risk (RR), with 95% confidence interval (95% C[illegible]nd p-value for each period variable. Statistical analyzes were univariate and multivariate in both models. The choice of the Negative Binomial Regression Model was based on the discrete nature of the length of stay count data. Since in this study the mean length of stay (11,96432) was lower than its variance (252,0617), the negative binomial regression was appropriate for modeling data dispersion and providing narrower confidence intervals, as well as allowing results interpretation in terms of relative risk (RR). The analyses were performed using the R Project for Statistical Computing software version 3.4.4.

This study was part of the project “Epidemiological, economic and care paths of high cost procedures in SUS: use of patient-centered database from the integration of health

information system records", approved by the Research and Ethics Committee of the Federal University de Minas Gerais, with Process number: CAAE 44121315.2.0000.5149.

Results

Table 1 presents the study population according to demographic and clinical characteristics. We included 417,477 patients who started cancer treatment by the SUS, between 2010 and 2014.

Table 1- Sociodemographic and Clinical Characteristics of the Cancer Patients Treatmented by Brazilian Public Health System, Brazil, 2010-2014.

	**W[illegible]LE PO[illegible]ATION**		**HOSPITALIZATION**				
			**NOT**		**YES**		
			n	(%)	n	(%)	
	n	(%)	275 416	(66.0)	142 061	(34.0)	*p* value
**GENDER**							**<0.05**
Male	166 626	(39.9)	113 895	(68.4)	52731	(31.6)	*
Female	250 851	(60.1)	161 521	(64.4)	89,330	(35.6)	*
**AGE**							
Medium [SD]	60.8	[13.6]	61.7	[13.5]	58.9	[13.8]	*
**AGE GROUP**							**<0.05**
19 – 24	1,031	(0.2)	438	(42.5)	593	(57.5)	*
25 – 39	28,652	(6.9)	16,240	(56.7)	12,412	(43.3)	*
40 – 59	155 129	(37.1)	97,054	(62.6)	58,075	(37.4)	*
60 – 79	201 336	(48.2)	138 890	(69.0)	62,446	(31.0)	*
≥ 80	31,329	(7.6)	22,794	(72.8)	8535	(27.2)	*
**REGION OF RESIDENCE**							**<0.05**
North	17,121	(4.1)	12,773	(74.6)	4348	(25.4)	*
Northeast	91,535	(21.9)	63,896	(69.8)	27,639	(30.2)	*
Southeast	198 249	(47.5)	131 623	(66.4)	66,626	(33.6)	*
South	84,828	(20.3)	50,662	(59.7)	34,166	(40.3)	*
Midwest	25,744	(6.2)	16,462	(63.9)	9282	(36.1)	*
**PRIMARY TUMOR LOCATION**							**<0.05**
Prostate	105 153	(25.2)	87,190	(82.9)	17,963	(17.1)	*
Breast	157 959	(37.8)	110 204	(69.8)	47,755	(30.2)	*
Cervix	42,837	(10.3)	27,934	(65.2)	14,903	(34.8)	*
Stomach	22,532	(5.4)	10,241	(45.5)	12,291	(54.5)	**
Lung and Bronchi	30,022	(7.2)	13,630	(45.4)	16,392	(54.6)	**
Colon and Rectum	58,974	(14.1)	25,713	(43.6)	33,261	(56.4)	*
**CANCER STAGE**							**<0.05**

0	19,449	(4,6)	13,809	(71.0)	5640	(29.0)	*
I	52,472	(12.6)	42689	(81.4)	9783	(18.6)	*
II	123 155	(29.5)	93,723	(76.1)	29,432	(23.9)	*
III	137 555	(32.9)	79,482	(57.8)	58,073	(42.2)	*
IV	84,846	(20.4)	45,713	(53.9)	39,133	(46.1)	*
**COMORBIDITIES**							**<0.05**
0	70,784	(16.9)	64,113	(90.6)	6671	(9,4)	*
1 – 3	180 044	(43.2)	126 539	(70.3)	53,505	(29.7)	*
> 3	166 649	(39.9)	84,764	(50.9)	81,885	(49.1)	*

* P <0.05 at the intersection with the other of the categories ** p = 0.88 at the intersection of the categories Primary Tumor Location Stomach and Lung and Bronchi

We observed a predominance of females (60,1%), aged 60-79 years and residents of the Southeast region. The hospitalization at least once within the first year of cancer treatment occurred in 34% of patients. The mean age of patients was lower among those hospitalized, 58.9 years. There was a decrease of the hospitalization frequency with increasing age. The largest difference of the hospitalized patient’s frequency was among residents of North (25.4%) and South (40.3%) regions. Women with breast cancer and men with prostate cancer together accounted for 63% of the study patients. However, the highest hospitalization frequency was registered in individuals with colon and rectum tumor. In the whole study population, 55.3% of patients started cancer treatment with extensive local tumor or extensive regional lymphatic invasion (stage III), or locally advanced or metastatic tumor (stage IV). The highest frequency of hospitalizations was found among patients with stage III (42.2%) and IV (46.1%). Most of the individuals (83.1%) had comorbidities at the cancer treatment start, and the highest hospitalization frequency was among those with 4 or more comorbidities.

We observed a mean of 2.1 hospitalizations per patient within the first year after treatment start and mean length of stay of 12 days. Most of the patients (65.9%) were emergency hospitalized for at least once during the period.

Non-oncology-specific surgical procedures for cancer treatment motivated the majority (32.1%) of hospitalizations, followed by the 'hospitalizations due to the clinical complications of cancer treatment' (24.4%). Oncological surgeries accounted for 23.2% of all hospitalization causes, while hospitalizations for other clinical causes occurred in 16.3% of cases. Finally, only 3.6% of hospitalizations were performed for the administration of radiotherapy or chemotherapy. Among patients hospitalized more than once within the first year after treatment start, 59% had at least one hospitalization to treat clinical cancer complications.

The final regression model for the hospitalization as outcome variable (Table 2) suggested that among patients who started treatment between 2010 and 2014, women were 16% less likely to be hospitalized (OR = 0.84; CI: 0.82-0.86), one-year of age increase reduced 2% the chance of hospitalization (OR = 0.98; CI: 0.98-0.99) and, regarding the patient's residence region, patients from all regions had higher chance of hospitalization compared to North region patients. Furthermore, those patients who live in the South region had the twice hospitalization chance when against North region patients (OR = 2.01; CI: 1.93-2.10).

Table 2- Logistic Regression Analysis of Hospitalization for Cancer Patients within the First Year After Treatment Start by Brazilian Public Health System, Brazil, 2010-2014.

	**Univariate**			**Multivariate**		
	OR	95%	*p*	OR	95%	*p*
Intercept	-	-	-	0.13	0.126 - 0.14	
**GENDER§**						
Female	1.19	1.17 - 1.21	*	0.84	0.82 - 0.86	*
**AGE**	0.98	0.98 - 0.98	*	0.98	0.98 - 0.99	*
**REGION OF RESIDENCE §**						
Northeast	1.27	1.22 -1.31	*	1.38	1.33 - 1.44	*
Southeast	1.48	1.43 -1.54	*	1.43	1.38 - 1.49	*
South	1.98	1.91 - 2.05	*	2.01	1.93 - 2.10	*
Midwest	1.65	1.58 -1.72	*	1.75	1.67 - 1.83	*
**PRIMARY TUMOR LOCATION§**						
Colon and Rectum	5.82	5.65 - 6.00	*	4.42	4.27 - 4.48	*
Stomach	5.84	5.70 - 5.97	*	4.41	4.29 - 4.53	*
Lung and Bronchi	6.28	6.11 - 6.46	*	4.35	4.21 - 4.48	*
Cervix	2.59	2.52 - 2.65	*	2.61	2.51 - 2.71	*
Breast	2.10	2.06 - 2.14	*	2.42	2.34 - 2.50	*
**CANCER STAGE§**						
I	0.56	0.54 - 0.58	*	0.56	0.54 - 0.58	*
II	0.76	0.74 - 0.79	*	0.78	0.75 - 0.81	*
III	1.78	1.73 - 1.84	*	1.55	1.50 - 1.61	*
IV	2.09	2.02 - 2.16	*	1.62	1.57 - 1.70	*
**COMORBIDITIES**	1.20	1.20 - 1.21	*	1.19	1.18 - 1.19	*

* P <0.001, respectively §categorias reference: male; North; prostate situ (0).

Regarding the primary tumor location, patients with tumors in the colon and rectum (OR = 4.42; CI: 4.27-4.48), stomach (OR = 4.41; CI: 4.29-4, 53) and lung (OR = 4.35; CI: 4.21-4.48) had higher chances of hospitalization. The hospitalization chance increased with the progress of tumor stage, being the patients with cancer stage IV more likely to be hospitalized in the order of 62% (OR = 1.62; CI: 1.57-1.70). Each occurrence of comorbidity increased by

19% (OR = 1.19; CI: 1.18-1.19) the chance of hospitalization within the first year after treatment start.

The area under the ROC curve for the logistic regression model after adjustment of the study variables (AUC measure), had a 0.76 coefficient, which demonstrates the good performance of the model.

Table 3 presents the estimates of multivariate analysis model, with the factors associated to the length of hospital stay within the first year after treatment start (significance level: $p \leq 0.05$). The demographic factors associated with the risks of higher length of stay were: to be male (RR = 0.98 for women; CI: 0.97-0.99); with younger ages, since each year of age reduced by 1% the risk of hospital stay (RR = 0.99; CI: 0.98-0.99); resident of the North region (RR≤ 0.93 for the other regions); with primary tumor location in the colon and rectum (RR = 1.37; CI: 1.35-1.40); and to present tumor metastasis, stage IV (RR = 1.04; CI: 1.04-1.06). Each comorbidity increased by 3% (RR = 1.03; CI: 1.02-1.03) the risk of higher length of hospital stay; and to be emergency hospitalized increased this risk by 28% (RR = 1.28; CI: 1.27-1.28).

Table 3- Negative Binomial Regression Analysis of Length of Hospital Stay for Cancer Patients within the First Year After Treatment Start by Brazilian Public Health System, Brazil, 2010-2014.

	**Univariate**			**Multivariate**		
	RR	95%	*p*	RR	95%	*p*
Intercept	-	-	-	8.08	7.72 - 8.46	< 0.001
**GENDER§**						
Female	0.76	0.75 - 0.77	*	0.98	0.97 - 0.99	0.032
**AGE**	0.99	0.98 - 0.99	*	0.99	0.98 - 0.99	*
**REGION OF RESIDENCE§**						
Northeast	0.94	0.90 - 0.97	*	0.93	0.93 - 0.98	*
Southeast	0.92	0.89 - 0.95	*	0.81	0.79 - 0.83	*
South	0.99	0.96 - 1.03	0.86	0.75	0.73 - 0.77	*
Midwest	0.80	0.77 - 0.83	*	0.66	0.64 - 0.68	*
**PRIMARY TUMOR LOCATION§**						
Colon and Rectum	1.53	1.50 - 1.56	*	1.37	1.35 - 1.40	*
Cervix	1.50	1.47 - 1.53	*	1.35	1.32 - 1.38	*
Stomach	1.48	1.45 - 1.52	*	1.27	1.25 - 1.30	*
Breast	0.58	0.57 - 0.59	*	0.68	0.66 - 0.69	*
Lung and Bronchi	1.36	1.33 - 1.38	*	1.22	1.20 - 1.25	*
**CANCER STAGE§**						
I	0.84	0.81 - 0.87	*	0.92	0.89 - 0.95	*
II	0.89	0.86 - 0.91	*	0.91	0.89 - 0.94	*
III	0.94	0.92 - 0.97	*	0.95	0.93 - 0.97	*
IV	1.24	1.21 - 1.28	*	1.04	1.01 - 1.06	*
**COMORBIDITIES**	1.06	1.05 - 1.06	*	1.03	1.02 - 1.03	*
**EMERGENCY HOSPITALIZATION**	1.32	1.32 - 1.33	*	1.28	1.27 - 1.28	*

* P <0.001Reference §categorias respectively: Male; North; prostate situ (0).

6. Discussion

This study evaluated the factors associated with hospitalization and length of hospital stay within the first year after treatment start, for a population of 417,477 Brazilian patients who underwent cancer treatment by the SUS, between 2010 and 2014, considering the types of cancer: breast, cervix, prostate, lung, stomach and colon and rectum. We observed that the occurrence of at least one hospitalization within the first year after treatment start occurred in 34% of patients. Several clinical characteristics were associated with hospitalization, such as: primary tumor location in lung, stomach, and colon and rectum; cancer stages III and IV; and higher number of comorbidities at treatment start. On the other hand, some demographic characteristics reduced the chance of hospitalization, namely: to be female, with older ages, and resident of the North region. Regarding the length of hospital stay, the same clinical variables above were predictors of a higher length of stay (primary tumor in colon and rectum; cancer stages III and IV; higher number of comorbidities at treatment start; and to be emergency hospitalized). However, in relation to demographic variables, we must emphasize that patients who lives in the North region had an increased risk of higher length of hospital stay.

Our population descriptive analysis revealed a predominance of female patients, residents of Southeast region, under breast cancer treatment, with older age at treatment start, with presence of comorbidities, and who started treatment with advanced cancer stages.

In recent years, Brazil expanded public policies for screening and diagnosis of cervical and breast cancers, then the female population within the health services has increased concerning the treatment of these cancer types [8]. The rise of prevalence of breast and prostate tumors is associated with the Brazilian demographic profile changes and the consequent population aging [2]. The epidemiological knowledge about the malignant tumors in study explains the observation of a large proportion of elderly people (60 and 79 years) in our results [2, 14]. Furthermore, approximately 80% of Brazilians aged 60 or more have at least one morbidity [3].

The distribution of the population by geographic region agrees with IBGE data, since the Southeast and Northeast regions contains the largest share of Brazilian population. However, regarding the hospitalized patients, the South region presented a higher proportion of individuals compared to the Northeast region, which may be correlated with the greater supply of hospital beds in the South region [14].

The great proportion of advanced tumor stages at treatment start may be explained by the late diagnosis of cancer, which is pointed as a serious public health issue in Brazil [1].

The frequent search of the cancer patients for emergency services due to acute conditions, which resulted in hospitalizations was also discussed by authors [5,9,17]. This is partly because, in cancer treatment, there would be a weak relationship between the patient and its healthcare team [4] or the contact with the referral professionals is limited.

The most cited pathologies (by ICD-10 codes) which lead cancer patients to seek emergency medical care, correspond to the procedure codes evaluated in this study regarding the patient's clinical complications, corroborating the findings of our research [5,15]. Some of these clinical complications in cancer patients are preventable by improving the quality of care during treatment [16].

The mean length of hospital stays of the study population, 12 days within the first year after treatment start, approached the results of other studies about cancer patients, even though with shorter follow-up time [12,17]. In particular, the inpatient stay in Japanese hospitals have been longer than observed in other developed countries, leading Japan to seek ways to reduce the hospital length of stay [17].

Regarding the evaluation of factors associated with hospitalization and length of hospital stay, other authors also demonstrated the predominance of male patients, associated with the presence of higher clinical severity and worse survival among men [17-19]. The adjustment of the variables for statistical analysis in this study leads to the assumption of other factors, and the need for further research to better comprehend the hospitalization patterns among male patients.

Our results regarding the age variable as reducer of the hospitalization chance and of risk for longer hospital stay for cancer patients differs from international studies that analyzed this association [17-20].

With the increase of patient's age, the pre-existing clinical conditions get worse, the drugs' toxicity is more exacerbated and slower biological response to changes in organic balance become more frequent [5,16]. Therefore, it is controversial the lower risk of hospitalization and length of hospital stay in older age cancer patients in Brazil, since the elderly represent constant demands in SUS emergency units [10].

About this matter, the current model of emergency care in Brazil and the expanse of the Emergency Care Units (UPAs) as an intermediate service between primary and hospital care, may support the explain about the lower chance of hospitalization and reduced length of hospital stay among elderly people. Patients admitted within the UPAs who have prescription to hospitalization should remain in the service for a maximum of 24 hours, while wait available

beds in the SUS hospitals. However, the insufficiency of hospital beds forces patients to stay longer periods in these units [10-11].

The lack of public hospital beds is so critical in Brazil that some managers account UPAs beds as hospital beds, and sometimes consider them as Intensive Care Units, even without the resources required for this kind of care. Many UPAs are not computerized and the length of stay in these services is not accounted by the Hospital Information System (SIH), since the payment of UPA's care provision is not made by Authorization of Hospital Inpatient (AIH) [11].

Regarding the patient's residence region, when analyzing the indicators of healthcare resources, we verify that the North region has the lowest supply of hospital beds in the country. Furthermore, it is observed a gradual annual decrease in the number of hospital beds in the North region, which reduced from 2.25 (in 1992) to 1.84 beds per 100,000 inhabitants (in 2010), fact that justifies the lower chance of hospitalization for patients on cancer treatment in the region [14].

The North region presents the worst indicators of cancer care in Brazil. All levels of care have insufficient resources, including hospital institutions. Also, the often lately diagnosis and delay to treatment start, even for cancers targeted by public policies, may give a comprehension about the higher length of hospital stay among patients of the region [1,8,16].

This regional inequality in Brazilian health system has also been evidenced by the analysis of palliative care implementation, which is poorly consolidated in the North region when compared, as example, with the Southeast. The most developed cities, such as São Paulo, have been pioneers in this strategy, leading to the early discharge of patients, which are allowed to cancer treatment by support of home care teams [2].

These qualified teams are capable to monitor cancer patients at home, in interface with the hospital staff, thus configuring one way to reduce the length of hospital stay [17]. The insufficiency of these kind of care and of material resources in the public system, which are needed to support patients with end stage disease, may give a comprehension about the reasons for seeking hospital services at end-of-life [4], thus occurring the expansion of length of stay and, consequently, of hospitalization expenses [7].

The higher risks of hospitalization registered to patients with tumors in stomach, lung, and colon and rectum are justified in the literature by the clinical aspects of these cancers, with emphasis on those associated with complications by surgical treatment [17, 19-20]. Other studies have also shown longer hospital stays for patients with colon and rectal cancer [19,20].

The clinical weakness of patients with metastatic tumors may require higher length of hospital stay for pain relief, hemodynamic instability or even emotional support of patients and their families since the cancer cure is not possible, argument that corroborates our study results [18,20].

Our observation that the increase of number of comorbidities has increased the risk of hospitalization and of higher length of hospital stay consist an intuitive situation. The stabilization of acute conditions from the comorbidities by itself may requires hospitalizations [6].

When the patient is hospitalized, the presence of comorbidities increases the risk of complications, what influences the planning of time for the hospital discharge [6,12,18].

The emergency hospitalization of cancer patients presupposes that these individuals present some serious pathologies that does not allow their outpatient control [5,9,15,17]. In most of the cases, emergency hospitalizations are assisted by general practitioners, without link to the patients' referral health team or without enough knowledge about oncology [16]. Under these conditions, the evaluation to define an accurate diagnosis and appropriate treatment may demand longer hospital stays for these patients [19].

The unavailability of data from patients who underwent only surgery as treatment, a modality that is often recommended for individuals with early stage tumors, represents a limitation of this study. This observation suggests the need for in-depth studies to a better comprehension of the evaluated subject. Otherwise, trough information of the factors associated to hospitalization and to the length of hospital stay of cancer patients, this study aims to stimulate future researches on this oncology area.

CONCLUSIONS

The public policies for cancer care in Brazil are subject of intensive analyses and debates. It is essential that emerges new perspectives about the cancer morbidity and mortality. For this, many efforts in defense of the SUS will be required to deal with the nowadays organizational structures of public health and its complexity. The current crisis of the hospital sector and the overload of emergency units pass by the historical insufficiency of the SUS funding.

The results of this study pointed out the importance of rethinking the effectiveness of cancer care, the dignity care for the elderly patients, the equity of access to hospitalization, the investment of resources in palliative care programs, and finally, how to mitigate the Brazilian regional inequalities in cancer care.

Acknowledgements

MLC, EIGA and FAA received research sponsorship from National Council of Technological and Scientific Development (CNPq), Brazil and The Minas Gerais State Research Foundation (FAPEMIG), Brazil. This study was part financed by the Coordination for the Improvement of Higher Education Personnel (CAPES) (Financial Code 001) and Fundação de Amparo à Pesquisa do Estado de Minas Gerais (FAPEMIG) grant number: APQ-03475-13.

REFERENCES

1. Oliveira MM, Malta DC, Guauche H, Moura L, Silva G. Estimated Number of People Diagnosed with Cancer in Brazil: Data from the National Health Survey, 2013. Rev. bras. epidemiol. 2015,18:146-157. Available from: http://www.scielo.br/scielo.php?script=sci_arttext&pid=S1415-790X2015000600146&lng=en. http://dx.doi.org/10.1590/1980-5497201500060013.

2. BRAZIL. Ministry of Health. Department of the Brazilian Public Health System (DATASUS). Brasilia. Available at: <http: // www.datasus.gov.br>.

3. BOCCOLINI CS, CAMARGO P. Morbidity and mortality from chronic diseases in Brazil: current and future situation. Rio de Janeiro: Oswaldo Cruz Foundation, 2016. 25 p. (Discussion texts, no. 22).

4. Seow H, Salam-White L, Bainbridge D. Community-based specialist palliative care teams and health system costs at end of life: a retrospective matched cohort study. CMAJ Open. 2019; 7(1):73-80. doi: 10.9778/cmajo.20180113.

5. Aprile G, Pisa FE, Follador A, Foltran L, De Pauli F, Mazzer M, Lutrino S, Sacco CS, Mansutti M, Fasola G. Unplanned presentations of cancer outpatients: a retrospective cohort study. Support Care Cancer. 2013; 2:397-404. doi: 10.1007/s00520-012-1524-6. PMID:22722887.

6. May P, Garrido MM, Aldridge MD et al. Prospective Cohort Study of Hospitalized Adults With Advanced Cancer: Associations Between Complications, Comorbidity, and Utilization. Journal of Hospital Medicine. 2017; 12(6): 407-413. DOI:10.12788/jhm.2745.

7. Park M, Song I. Medical care costs of cancer in the last year of life using national health insurance data in Korea. 2018; PLoS ONE 13(6): e0197891. https://doi.org/10.1371/journal. pone.0197891.
8. Santos MS, Oliveira MM, Andrade SA, Nunes L, Malta DC, Moura L.Non-communicable chronic disease hospital morbidity trends in Brazil, 2002-2012. Epidemiol.

Serv. Saúde. 2015; 24:389-398. doi:10.5123/s1679-49742015000300005. PMID:16794974.

9. Whitney RL, Bell JF, Tancredi DJ, Romano PS, Bold RJ, Joseph JG. Hospitalization Rates and Predictors of Rehospitalization Among Individuals With Advanced Cancer in the Year After Diagnosis. J Clin Oncol. 2017; 35:3610-3617. DOI: https://doi.org/10.1200/JCO. 2017.72.4963

10. Scolari GAS, Rissardo LK, Baldissera VDA, Carreira L. Emergency care units and dimensions of accessibility to health care for the elderly. Rev Bras Enferm. 2018;71(suppl 2):811-7. DOI: http://dx.doi.org/10.1590/0034-7167-2017-0440.

11. O'Dwyer G, Konder MT, Reciputti LP, Lopes MGM, Agostinho DF, Alves GF. The implementation process of the emergency units in Brazil. Journal of Public Health. 2017;51:125. DOI: https://doi.org/10.11606/S1518-8787.2017051000072.

12. Farjah F, Lou F, Rusch VW, Rizk NP. The Quality Metric Prolonged Length of Stay Misses Clinically Important Adverse Events. Ann Thorac Surg. 2012; 94:881– 888. doi:10.1016/j.athoracsur.2012.04.082. PMID: 00034975.

13. Guerra Junior AA, Pereira RG, Andrade EIG et al. Building the National Database of Health Centred on the Individual: Administrative and Epidemiological Record Linkage - Brazil, 2000-2015. International Journal of Population Data Science. 2018; 3:3:20. DOI: https://doi.org/10.23889/ijpds.v3i1.446.

14. BRAZILIAN INSTITUTE OF GEOGRAPHY AND STATISTICS. A health Panorama in Brazil: access and use of services, health conditions and risk factors and health protection: 2008. Rio de Janeiro: IBGE, 2010. 256 p.

15. Tamang S, Patel MI, Blayney DW et al. Detecting Unplanned Care From Clinician Notes in Electronic Health Records. Journal of Oncology Practice. 2015; 11(3):313-319.DOI: 10.1200/JOP.2014.002741 . PMID: 25980019.

16. Pugh A, Rogerson H, Cosh H, Hart J, Williams H, Jenkins C. Acute oncology: a developing sub-specialty. Br J Nurs. 2015, 24(16):S18-25. doi: 10.12968/bjon.2015.24.Sup16.S18.

17. Kunisawa S, Fushimi K, Imanaka Y. Reducing Length of Hospital Stay Does Not Increase Readmission Rates in Early-Stage Gastric, Colon, and Lung Cancer Surgical Cases in Japanese Acute Care Hospitals. PLOS ONE. 2016; 11: 1-10. doi:10.1371/journal.pone.0166269.

18. O'Connor NR, Hu R, Harris PS, Ache K, Casarett DJ. Hospice admissions for cancer in the final days of life: independent predictors and implications for quality measures. J Clin Oncol. 2014, 32(28):3184-9. doi: 10.1200/JCO.2014.55.8817.

19. Kelly M, Sharp L, Dwane F, Kelleher T, Comber H. Factors predicting hospital length-of-stay and readmission after colorectal resection: a population-based study of elective and emergency admissions. BMC Health Services Research. 2012, 12:77 https://doi.org/10.1186/1472-6963-12-77.

20. Daw-Shyong P, I-Cheng L, Hon-Yi S et al. Incidence trends and predictors for cost and average lengths of stay in colorectal cancer surgery. World J Gastroenterol. 2014, 20(2): 532-538. doi:10.3748/wjg.v20.i2.532.
